# Genome-Wide Characterization and Expression Analysis Provide Basis to the Biological Function of Cotton FBA Genes

**DOI:** 10.3389/fpls.2021.696698

**Published:** 2021-08-19

**Authors:** Zhong-Qing Li, Yao Zhang, He Li, Ting-Ting Su, Cheng-Gong Liu, Zi-Chao Han, Ai-Ying Wang, Jian-Bo Zhu

**Affiliations:** Key Laboratory of Agricultural Biotechnology, College of Life Science, Shihezi University, Shihezi, China

**Keywords:** cotton, Calvin-Benson cycle, evolution, expression profiles, FBA

## Abstract

Fructose-1,6-biphosphate aldolase (FBA) is a multifunctional enzyme in plants, which participates in the process of Calvin-Benson cycle, glycolysis and gluconeogenesis. Despite the importance of *FBA* genes in regulating plant growth, development and abiotic stress responses, little is known about their roles in cotton. In the present study, we performed a genome-wide identification and characterization of *FBAs* in *Gossypium hirsutum*. Totally seventeen *GhFBA* genes were identified. According to the analysis of functional domain, phylogenetic relationship, and gene structure, *GhFBA* genes were classified into two subgroups. Furthermore, nine *GhFBAs* were predicted to be in chloroplast and eight were located in cytoplasm. Moreover, the promoter prediction showed a variety of abiotic stresses and phytohormone related *cis*-acting elements exist in the 2k up-stream region of *GhFBA*. And the evolutionary characteristics of cotton *FBA* genes were clearly presented by synteny analysis. Moreover, the results of transcriptome and qRT-PCR analysis showed that the expression of *GhFBAs* were related to the tissue distribution, and further analysis suggested that *GhFBAs* could respond to various abiotic stress and phytohormonal treatments. Overall, our systematic analysis of *GhFBA* genes would not only provide a basis for the understanding of the evolution of *GhFBAs*, but also found a foundation for the further function analysis of *GhFBAs* to improve cotton yield and environmental adaptability.

## Introduction

It has been proved that photosynthesis is the physiological basis in crop yield formation, and the intensity of photosynthesis determines the level of crop production. Ribulose-1,5-bisphosphate carboxylase/oxygenase (Rubisco), as the catalyticase in the first step of Calvin-Benson cycle with low catalytic efficiency, was always thought to be the main rate-limiting mechanism of photosynthesis under normal growth conditions (Portis, 2003). However, according to the analysis of antisense transgenic plants with reduced rubisco activity, some researches suggested that rubisco is not the only factor that control photosynthesis rate (Hudson et al., 1992; Stitt and Hurry, 2002), the enzymes in ribulose-1,5-bisphosphate (RuBP) regeneration pathway have significantly higher control coefficients than Rubisco in photosynthetic carbon flow (Raines, 2003; Cai et al., 2018).

Fructose-1,6-bisphosphate aldolase (FBA; EC 4.1.2.13) constitute a vital part of RuBP regeneration, it reversibly catalyzes the synthesis of fructose-1,6-diphosphate (FBP) from glyceraldehyde-3-phosphate (G3P) and dihydroxyacetone phosphate (DHAP). Additionally, FBA also catalyzes the synthesis of sedoheptulose 1,7-bisphosphate (SBP) from DHAP and erythrose 4-phosphate (E4P) (Flechner et al., 1999). And evidences showed that *FBA* might play an important role in the control of carbon metabolism rate and the regeneration of RuBP in Calvin-Benson cycle (Haake et al., 1998).

*FBAs* could be broadly classified into two classes according to their catalytic mechanisms and evolutionary origin (Marsh and Lebherz, 1992; Nakahara et al., 2003). The catalytic activity of class-I *FBAs* are not inhibited by ethylene diamine tetraacetic acid (EDTA) or affected by potassium ions, and class-I *FBAs* are most found in bacteria, animals and plants. While the enzyme activity of class-II subgroup is affected by EDTA and the members usually occur in bacteria, yeast, fungi and some higher plants (Penhoet et al., 1967; Tolan et al., 1987; Henze et al., 1998). In higher plants, the *FBA* genes were located in cytosolic (c*FBA*) and chloroplast/plastid (cp*FBA*) (Lebherz and Rutter, 1969; Bukowiecki and Anderson, 1974). Both c*FBAs* and cp*FBAs* are nuclear-encoded genes and play vital roles in carbohydrate metabolism (Anderson et al., 1995).

Hitherto, a number of works have been done to analyze the characteristics of *FBA* gene family in several plant, including *A. thaliana* (*AtFBA1-8*) (Lu et al., 2012), *O. sativa* (*OsFBA1-7*), *S. lycopersicum* (*SlFBA1-8*) (Cai et al., 2016), *B. napus* (*BnaFBA1-22*) (Zhao et al., 2019) and *T. aestivum* (*TaFBA1-21*) (Lv et al., 2017). *FBAs* have been shown to be related to diverse physiological and biochemical processes in plants. Over-expressing *AtFBA* could improved plant growth in transgenic tobacco (Uematsu et al., 2012). While decreased the expression level of *SlFBA7* would significantly reduce the biomass of transgenic tomato (Cai et al., 2018). Furthermore, *FBA* genes were also reported to be involved in signal transduction (Oelze et al., 2014; Zhang et al., 2014), secondary metabolism (Zeng et al., 2014) and resistance to abiotic stresses (Michelis and Gepstein, 2000; Yamada et al., 2000; Oztur et al., 2002; Larkindale and Vierling, 2008; Purev et al., 2008; Xue et al., 2008; Fan et al., 2009; Lu et al., 2012). Moreover, the fact that *FBAs* appear in the nucleus indicated *FBA* genes might function as transcriptors in regulating gene expression directly (Páez-Valencia et al., 2008). All these results indicated that *FBA* genes hold tremendous potentials for genetic engineering to improve the crop yield and stress resistance.

Cotton is an important economic crops that provide textile and oil materials worldwide (Huang et al., 2015). There are more than 50 species in the *Gossypium genus* (Wendel and Cronn, 2003), including six tetraploid (2n = 4×) species and 46 diploid (2n = 2×) species. Due to its high yield and high-quality fiber, *G. hirsutum* is the most widely cultivated among all cotton species. However, some abiotic stresses, including temperature, drought and salt, are all restrictions on cotton growth, fiber quality and yield (Yang et al., 2012; Gu et al., 2018; Li, 2018; Sun, 2019). As mentioned before, *FBA* genes play key roles in photosynthetic carbon flow and stress resistance, but only a few researches about *FBA* in cotton have been reported up to now (Xu et al., 2013). The whole-genome sequencing of *G. raimondii*, *G. arboreum* and *G. hirsutum* provided an opportunity to have a novel insights into the *FBA* family at genome-wide level (Paterson et al., 2012; Li et al., 2014; Yang et al., 2019).

In present study, we systematically identified 17 putative *FBA* genes in *G.hirsutum*, then the phylogenetic relationships, gene characteristics, structures, and chromosomal distribution of the identified *FBA* genes were further analyzed in detail. Additionally, the expression of *FBAs* in different tissues and in response to different stresses and different phytohormones were further analyzed. This research would supply a valuable reference for function analysis of *FBA* genes in cotton and other species.

## Materials and Methods

### Identification of Cotton *FBA* Sequences

The genome sequences and annotation files of *G. arboreum* (Du et al., 2018), *G. raimondii* (Paterson et al., 2012), and *G. hirsutum* (Yang et al., 2019) were downloaded from CottonFGD.^1^ To identify the FBA in three cotton species, the Hidden Markov Model files corresponding to the Glycolytic domain (PF00274) and fructose-bisphosphate aldolase class-II domain (PF01116) were downloaded from Pfam protein family database.^2^ Then the *FBA* genes were extracted from the three cotton genome database by using HMMER (version 3.0) with default parameters, the isoforms were removed manually. For the further confirmation of *FBA* members, all the candidate *FBAs* genes were submitted to the CDD database and SMART database^3^ for the further examined.

### Sequence Analysis

The ExPASy website^4^ was used to compute the length of sequences, molecular weights (MW) and isoelectric point (pI) of cotton *FBA* genes. The coding sequences (CDS) and the corresponding full-length DNA sequences were used to predict the structures of *FBA* genes through the online bioinformatics tool Gene Structure Display Server (GSDS^5^). And the subcellular localization of cotton *FBA* genes were predicted by WoLF PSORT.^6^ Then the MEME website^7^ was used to identify the conserved motifs of cotton *FBA* genes. To obtain the *cis*-acting elements information in *GhFBA* promoters, 2-kb upstream sequences of each cotton *FBA* genes were intercepted and submitted to PlantCARE database for further analysis.

### Multiple Alignments and Phylogenetic Analysis

ClustaW was used to align the putative *GhFBA* amino acid sequences with default parameters, and then MEGA7.0 (Tamura et al., 2013) was employed to construct phylogenetic trees using Neighbor-Joining (NJ) method with 1000 bootstrap replications. The information of *FBA* proteins from Arabidopsis, tomato (Cai, 2017), wheat and rice were obtained according to the description of related reporters and the sequences were downloaded from TAIR and NCBI database.

### Chromosome Localization and Gene Duplication Analysis

The physical location information of *GhFBA* genes in the chromosomes were obtained from the genome annotation files of three species. And the duplication events within the subgenome were detected by Multiple Collinearity Scan toolkit (MCScanX) (Wang et al., 2012) with default settings. Then the TBtools^8^ was used to exhibit the chromosomes distribution and the synteny relationship of orthologous *FBA* genes that identified from *G. raimondii*, *G. arboreum*, and *G. hirsutum*. Furthermore, KaKs Calculator 2.0 was employed to estimate the non-synonymous (ka) and synonymous (ks) substitution rates of the duplicated cotton *FBA* genes.

### Expression Analysis of *GhFBA* Genes

The public available transcriptome data of *G. hirsutum* in different tissues and in respond to different stress were downloaded from CottonFGD database (BioProject ID: PRJNA49062) (Hu et al., 2019). And Transcripts Per Kilobase of exon model per Million mapped reads (TPM) was used to quantify gene expression. Then the TPM values were perform treatment of log_2_(TPM + 1) to construct the heatmap by TBtools.

### Expression Profile Analysis of *GhFBAs* in Response to Distinct Treatments

To validate the accuracy and authenticity of the transcriptome data and have a further understanding of the expression patterns of *GhFBA* genes in response to different phytohormonal treatments, eight *GhFBA* genes were selected for further qRT-PCR analysis. Full shape and disease-free seeds were selected and planted in pots, the greenhouse conditions were set at 28°C with 16 h light/8 h dark cycle. After 4 weeks cultivation, plants at three-leafs were selected for different treatments. The plants were treated with ABA (200 μM), ethylene (400 μM), MeJA (2 mM), SA (2 mM), 4°C, Nacl (200 mM) and PEG (20% *w/v*) for 1, 3, 6, 12, and 24 h, all selected samples were quickly frozen in liquid nitrogen and preserved at −80°C for subsequent analysis.

Total RNA of the samples was extracted using Plant RNA miniprep kit (Polysaccharides & Polyphenolics-rich, Biomiga, United States, R8611). Then the RevertAid First Strand cDNA Synthesis Kit (Thermo, United States, K1662) was used to synthesize the first strand of cDNA from the high quality DNA-free RNA. qRT-PCR was performed by using Roche LightCycler^®^ 480 instrument with SYBR Green (Transgen, China, AQ601). The qRT-PCR reaction conditions were as follows: 95°C for 5 min; 40 cycles of 95°C/15 s, 60°C/20 s, 72°C/30 s. Every treatments contained three three biological replications, and each analysis was repeated by three times. The date from qRT-PCR was analyzed using 2^–△△Ct^ method and the expression levels were represented by the mean values of the three replicates. Student’s *t* test was carried out to determine whether the changes in gene expression were significant. And sequences of the primers used in this study were shown in detail in Supplementary Table 1.

### Subcellular Location of *GhFBAs*

To verify the results of subcellular localization predication, the coding open reading frame (ORF) sequences without terminate codon of 7 *GhFBA* genes were cloned into *pCambia1300-eGFP* vector. The recombinant vectors *pCambia1300-GhFBAs-eGFP* and the empty vector *pCambia1300-eGFP* were transformed into *Agrobacterium tumefaciens* GV3101, then the *Agrobacterium tumefaciens* contained vectors were injected into three- to four-week old *Nicotiana benthamiana* leaves. After 2 days cultured in dark environment, the *GFP* signals were monitored using a laser scanning microscope (NIKON, C2^+^, Japan).

### Pearson Correlation Analysis

Based on the transcriptome data, the Pearson correlation coefficients (PCCs) and *p*-value of the expression levels of *GhFBA* gene pairs were calculated by SPSS (Version 26.0). The heatmap of correlations was generated by Tbtools. And the co-regulatory networks were constructed by Cytoscape (version 3.8.2) based on the PCCs of *GhFBAs* genes pairs with *p*-value <0.05.

## Results

### Identification of *FBA* Genes in Cotton

**FIGURE 1:**
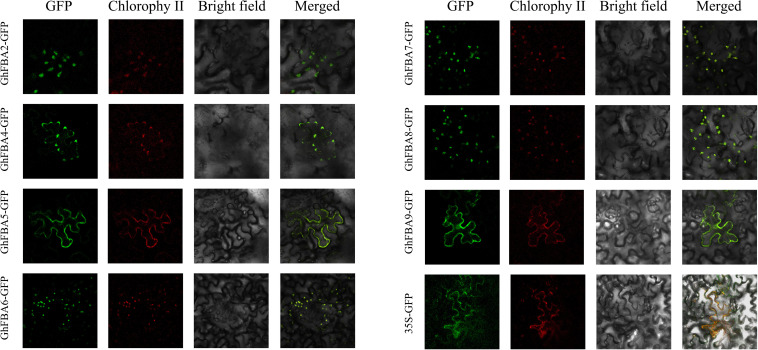
Subcellular location of *GhFBAs* in tobacco epidermal cells. *GhFBAs-eGFP* and empty control vector (35S-*eGFP*) were transiently expressed in tobacco epidermal cells. And the fluorescent signal was collected by confocal microscope.

Totally 17 putative *FBA* gene sequences were obtained *G. hirsutum* genome dataset (Table 1), furthermore, 9 *GaFBAs* amd 9 *GrFBAs* were obtained from *G. arboreum* and *G. raimondii* with the same methods (Supplementary Table 2). Based on the sequences information, gene characteristics, including the length of CDS and protein sequences, the MW and pI of *FBA* proteins, and the potential subcellular localization were all analyzed. As results, the length of deduced proteins of all *FBAs* ranged from 177 (*GhFBA2*) amino acid (aa) to 1373 (*GhFBA3*) aa. The highest MW was 147.87 kDa of *GhFBA3* protein and the lowest one was 19.57kDa of *GhFBA2*. The grand average of hydropathy (GRAVY) ranged from −0.244 (*GhFBA6*) to 0.096 (*GhFBA11*). The result of subcellular localization prediction showed that nine *GhFBAs* proteins (*GhFBA2/3/6/7/8/11/14/15/17*) were predicted to be located in chloroplast, and the other 8 *GhFBAs* proteins (*GhFBA1/4/5/9/10/12/13/16*) were located in cytoplasm (Table 1). Furthermore, 5 *GaFBAs* and 5 *GrFBAs* were predicted to be located in chloroplast, four *GaFBAs* and four *GrFBAs* were predicted to be cytoplasm-localized, respectively (Supplementary Table 2). To verify the predicted results of subcellular location, 7 *GhFBA* genes were selected and fused with *eGFP* protein to assay their presence in tobacco leaf cell. As shown in Figure 1, *GhFBA2/6/7/8* were located in chloroplast, and *GhFBA 4/5/9* were located in cytoplasm. This result was consistent with the prediction.

**TABLE 1:** Characteristics information of FBA family genes in *G.hirsutum*.

Gene name	Locus name	Gene location^1^	Transcript length (bp)	Protein length(aa)	PI	MW (kDa)	GRAVY	Subcellular localization
*GhFBA1*	Gh_A01G234900.1	A01: 113549843-113551804: −	1,077	358	6.56	38.53	–0.107	Cytoplasm
*GhFBA2*	Gh_A02G200600.1	A02: 104986095-104987421: +	534	177	4.84	19.57	–0.241	Chloroplast
*GhFBA3*	Gh_A03G110000.1	A03: 45882052-45931333: −	4,379	1,373	6.29	147.87	0.092	Chloroplast
*GhFBA4*	Gh_A04G083000.1	A04: 54215091-54217168: +	1,412	358	7	38.65	–0.189	Cytoplasm
*GhFBA5*	Gh_A04G083400.1	A04: 54950062-54952352: +	1,489	358	5.95	38.58	–0.161	Cytoplasm
*GhFBA6*	Gh_A05G326000.1	A05: 75119125-75122151: +	1,601	394	8.95	42.84	–0.244	Chloroplast
*GhFBA7*	Gh_A12G065500.1	A12: 14193161-14195150: −	1,441	396	8.44	42.85	–0.163	Chloroplast
*GhFBA8*	Gh_A13G029000.1	A13: 3158667-3167383: −	1,360	397	8.44	42.96	–0.158	Chloroplast
*GhFBA9*	Gh_A13G148800.1	A13: 89373702-89375399: −	1,390	357	7.56	38.43	–0.18	Cytoplasm
*GhFBA10*	Gh_D01G229000.1	D01: 62395873-62398103: −	1,347	358	6.56	38.55	–0.113	Cytoplasm
*GhFBA11*	Gh_D02G136400.1	D02: 41762499-41806602: −	4,059	1,352	6.19	145.20	0.096	Chloroplast
*GhFBA12*	Gh_D04G119500.1	D04: 36273396-36276196: −	1,993	358	6.48	38.75	–0.168	Cytoplasm
*GhFBA13*	Gh_D04G119600.1	D04: 36446861-36448986: −	2,126	388	6.91	42.04	–0.076	Cytoplasm
*GhFBA14*	Gh_D12G063100.1	D12: 10811052-10813066: −	1,460	396	8.63	42.85	–0.164	Chloroplast
*GhFBA15*	Gh_D13G030700.1	D13: 2819815-2828582: −	1,528	397	8.09	42.91	–0.143	Chloroplast
*GhFBA16*	Gh_D13G149800.1	D13: 46162956-46164514: −	1,252	357	6.85	38.27	–0.161	Cytoplasm
*GhFBA17*	Gh_Contig00785_ERROPOS 280090G000200.1	Contig00785_ERROPOS280090: 100384-103005: +	1,146	381	8.19	41.46	–0.226	Chloroplast

*^1^Chromosome: start position-end position: strand, (−) means antisense strand of chromosome, (+) means positive-sense strand of chromosome.*

### Phylogenetic Analysis of *GhFBA* Gene Family

**FIGURE 2:**
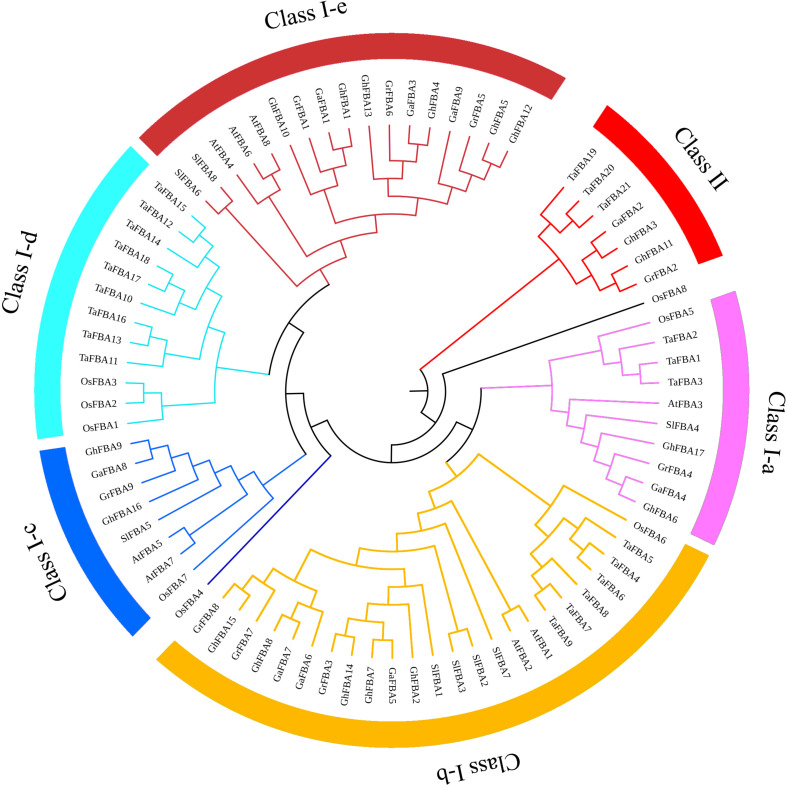
An unrooted phylogenetic tree representing relationships among FBA proteins from *G. hirsutum (Gh)*, *G. arboreum (Ga)*, *G. raimondii (Gr)*, *A. thaliana (At)*, *T. aestivum (Ta), O. sativa (Os)* and *S. lycopersicum (Sl)*. The different colors arcs indicate different classes or subclasses. Clustal W was used to align the sequences, MEGA7.0 was used to construct phylogenetic trees with neighbor-joining method.

To investigate the molecular phylogenetic relationships of the members in *GhFBA* gene family, totally 80 *FBA* genes from *G. hirsutum* (Gh), *G. arboreum* (Ga), *G. raimondii* (Gr), *A. thaliana* (At), *S. lycopersicum* (Sl), *T. aestivum* (Ta) and *O. sativa* (Os) were extracted to construct an unrooted phylogenetic tree. Based on the phylogenetic relationships of the selected genes, all the tested *FBAs* could be broadly classified into two major classes (Figure 2). Moreover, the class-I group was further divided into five subclasses (ClassIa-e). Among 17 *GhFBA* proteins, 2 belong to class-Ia, 5 belong to class-Ib, 2 belong to class-Ic, 6 belong to class-Ie and 2 belong to class-II. Furthermore, the absence of dicotyledonous *FBA* genes in group class-Id (Figure 2) indicated the evolutionary differences between monocotyledons and dicotyledons.

### Exon/Intron Organization and Motif Composition Analysis of Cotton *FBA* Genes

**FIGURE 3:**
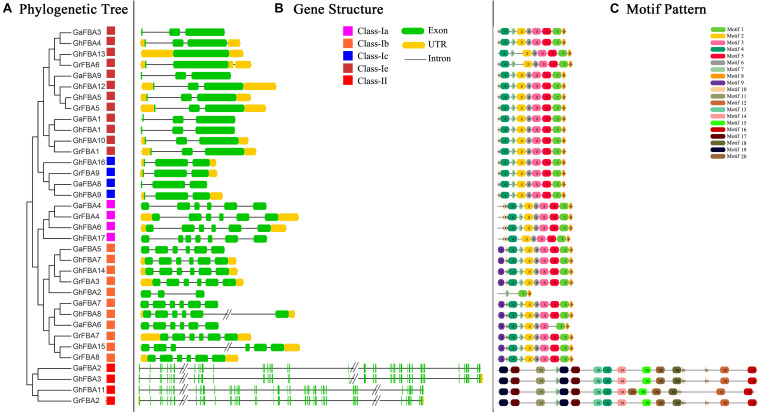
Phylogenetic relationships, gene structure and architecture of conserved protein motifs in *FBA* genes from *G. hirsutum (Gh)*, *G. arboreum (Ga)* and *G. raimondii (Gr).* **(A)** The phylogenetic tree of all identified cotton FBA genes. Full-length protein sequences of FBA genes were used to generate the phylogenetic tree, and the different color squares represent phylogenetic subclasses. **(B)** Gene structure features of cotton *FBA* genes. Green boxes indicate exons, black lines indicate introns and yellow boxes indicate 3′ and 5′ untranslated regions. **(C)** The motif architecture of cotton *FBA* proteins.

In order to provide enough proof for the phylogenetic analysis, we carried out a comparison of the predicted CDS of all identified *FBA* genes in *G. hirsutum*, *G. arboreum* and *G. raimondii*. As shown in Figure 3B, the distribution of exons and introns of cotton *FBA* genes was variable, the number of exons and the length of sequences were widely ranged between different subclasses. The number of exons ranged from one to six in class-I, and *GhFBA13* was the gene with only one exon, the genes in class-II all have 42 exons. Moreover, the members in class-Ia and class-Ib have more exons than the members in class-Ic and class-Ie. Further analysis showed that the members within the same subclasses usually shared similar structures, for example, except for *GhFBA2*, other genes in class-Ib subclass all contained five introns and six exons. Additionally, the *FBA* genes in *G. hirsutum* exhibited the same gene structures with its diploid parent.

Besides the study of gene structure features, predicted amino acid sequences of cotton *FBA* proteins were submitted to the MEME website for architecture analysis. As shown in Figure 3C, the differences of motifs between class-I and class-II were significant. Motif 2, motif 3, motif 5 and motif 8 were unique to class-I, while motif 15 motif 16 and motif 19 were specific to class-II. Except for the special motifs, some motifs were widely distributed in class-I and class-II, such as motif 7, motif 10 and motif 4. Additionally, within the same subclasses, the types and distribution of motifs shared high similarities, indicating that the protein architecture of cotton *FBA* genes was highly conserved within a specific subclass. According to the previous studies, *FBA* genes were related to diverse physiological and biochemical processes in plants, so further and deeper researches are needed to be carried out to expound the function of these conserved motifs.

### *Cis-*Acting Elements Analysis in the Putative Promoter Regions of *GhFBA* Genes

**FIGURE 4:**
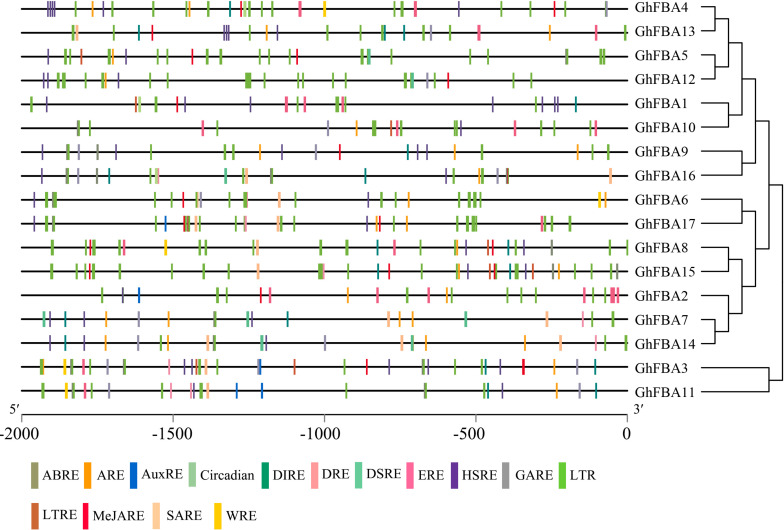
Analysis of the responsive *cis*-acting elements in *GhFBA* genes promoter regions. The 2-kb sequences of *GhFBA* gene promoter regions were extracted and analyzed, and different *cis*-acting elements were color-coded in specific colors.

The *cis*-acting elements within promoter regions of *GhFBA* genes were analyzed in this study (Supplementary Table 3). As shown in Figure 4, various *cis*-acting regulatory elements were detected in the promoter regions of *GhFBA* genes. Phytohormone responsive elements, such as MeJARE (MeJA-responsive element), ABRE (abscisic acid-responsive element), SARE (salicylic acid-responsive element) were included in the promoter regions (Figure 4), suggesting that the expression of *GhFBA* genes might be regulated by multiple phytohormones. Additionally, some stress-related *cis*-acting elements, like DSRE (drought and stress-responsive element), LTRE (low-temperature-responsive element) and HSRE (heat stress-responsive element) were also found in the *GhFBA* gene promoter regions (Table 2), these results indicated that *GhFBA* genes might be closely related to the responses to multiple abiotic stresses. Moreover, two phytohormone-related elements (ABRE, MeJARE) and a stress-responsive element (HSRE) were frequently detected in the putative promoters of *GhFBA* genes. Notably, each *GhFBAs* contain multiple copies of LRE (light-responsive element), suggesting that *GhFBA* genes was an important component of light response in *G. hirsutum*.

**TABLE 2:** Response-related *cis*-acting elements in the promoter regions of *GhFBA* genes.

Genes	*Cis*-acting elements														
	ABRE	ARE	AuxRE	Circadian	DRE	DSRE	DIRE	ERE	GARE	HSRE	LRE	LTRE	MeJARE	SARE	WRE
*GhFBA1*				1			1	4		7	7	1	2		
*GhFBA2*		2	1					7		1	11		2		
*GhFBA3*	3	2	1		1		2	1	3	5	12	2	6	1	2
*GhFBA4*		2		1			1	4	1	6	14		4		2
*GhFBA5*	1	1				1				2	19	1	4		
*GhFBA6*	1	2			1				1	2	22		2	2	1
*GhFBA7*	3	4			1	3	2		1	3	4			2	
*GhFBA8*	1	1					2	2		2	17	1	4	1	1
*GhFBA9*	3	3					1		2	5	9		2		
*GhFBA10*	1	1						4	1	1	11	1			
*GhFBA11*	2	1	2		2		2	1	2	2	11			1	2
*GhFBA12*		1				1			1	3	20		2		
*GhFBA13*		2					3	3	1	4	9		2	1	
*GhFBA14*	3	5			1	2	1		2	3	6			3	
*GhFBA15*	3	2			1		2			2	20	2	6	1	
*GhFBA16*	5	1		1	1	1	2		2	2	9		2	2	
*GhFBA17*	3	2	1		1			1		2	22		6	2	
Total	29	32	5	3	9	8	19	27	17	52	223	8	44	16	8

*ABRE, Abscisic acid-responsive element; ARE, Anoxic-responsive element; AuxRE, Auxin-responsive element; Circadian, Circadian-responsive element; DRE, Damage-responsive element; DSRE, Defense-and stress-responsive element; DIRE, Drought-responsive element; ERE, Ethylene-responsive element; GARE, Gibberellin-responsive element; HSRE, Heat stress-responsive element; LRE, Light-responsive element; LTRE, Low-temperature-responsive element; MeJARE, MeJA-responsive element; SARE, Salicylic acid-responsive element; WRE, Wound-responsive element.*

### Chromosomal Distribution and Synteny Analysis of *GhFBA* Genes

**FIGURE 5:**
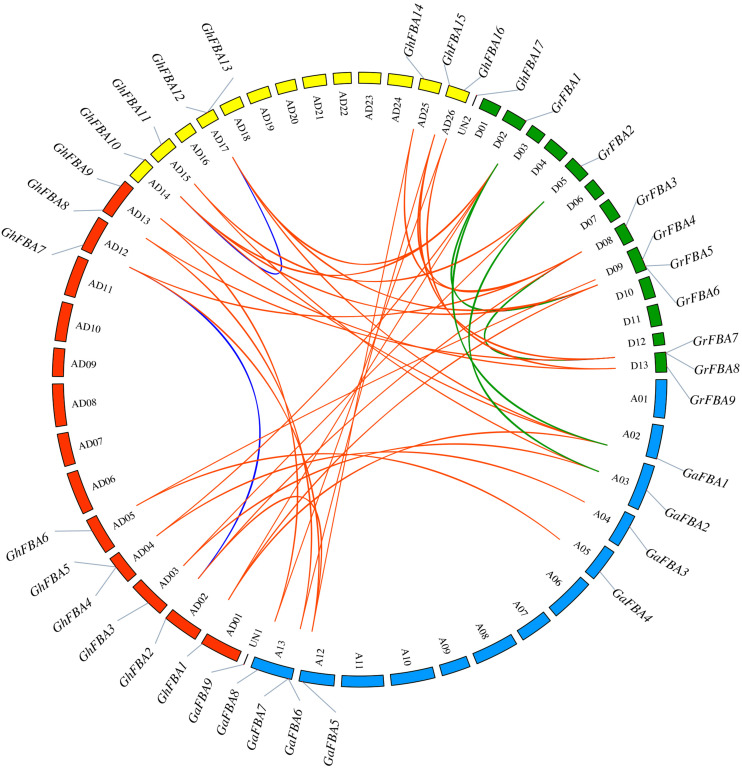
Chromosomal distribution and collinear correlations of FBA members of *G. hirsutum (Gh)*, *G. arboreum (Ga)* and *G. raimondii (Gr).* The chromosome number is indicated by the alphanumeric codes within the circle, UN1 and UN2 represent the scaffold that contain FBA genes in *G. arboreum* and *G. hirsutum*, respectively. The blue lines indicate the duplicated FBA gene pairs within A subgenome and D subgenome of *G. hirsutum*, the orange lines represent the syntenic FBA gene pairs between *G. hirsutum* and other species, the green lines represent the syntenic FBA gene pairs between *G. arboreum* and *G. raimondii.*

In order to have a further investigation into the evolution of *GhFBA* genes, the gene distribution in chromosomes and duplication events were analyzed. As shown in Figure 5, *GhFBA1-16* were distributed in 12 chromosomes, and *GhFBA17* was located on an unattributed contig (UN2). The number of *GhFBAs* in each chromosome ranged from 1 to 2, chromosome AD04, AD13, AD17 and AD26 contained two *GhFBA* genes. Additionally, 9 *GhFBA* genes were distributed over seven A subgenome chromosomes of *G. hirsutum* and 7 genes were located on five D subgenome chromosomes. Besides, no evidence showed that there was a positive correlation between the gene number and the length of chromosomes.

In the A subgenome of *G. hirsutum*, one segmental duplication event with two genes *GhFBA2/GhFBA7* was identified, and in the D subgenome a segmental duplication event and a tandem duplication event were identified (Supplementary Table 4). Moreover, for the sake of a better understanding of the evolutionary mechanism of *GhFBA* gene family, syntenic gene analysis was constructed among *G. hirsutum*, *G. arboreum* and *G. raimondii*. As results, 13 orthologous gene pairs were found between *G. hirsutum* and *G. arboreum*, 16 gene pairs between *G. hirsutum* and *G. raimondii*, 3 gene pairs between *G. arboreum* and *G. raimondii* (Figure 5). Additionally, the Ka/Ks ratios of all segmental and tandem duplicated *GhFBA* gene pairs, and the orthologous *FBA* gene pairs were lower than 1 (Supplementary Table 5), suggesting that the purifying selection may play an essential role during the *GhFBA* gene family evolution.

### Expression Profiles of *GhFBA* Genes in Different Tissues

**FIGURE 6:**
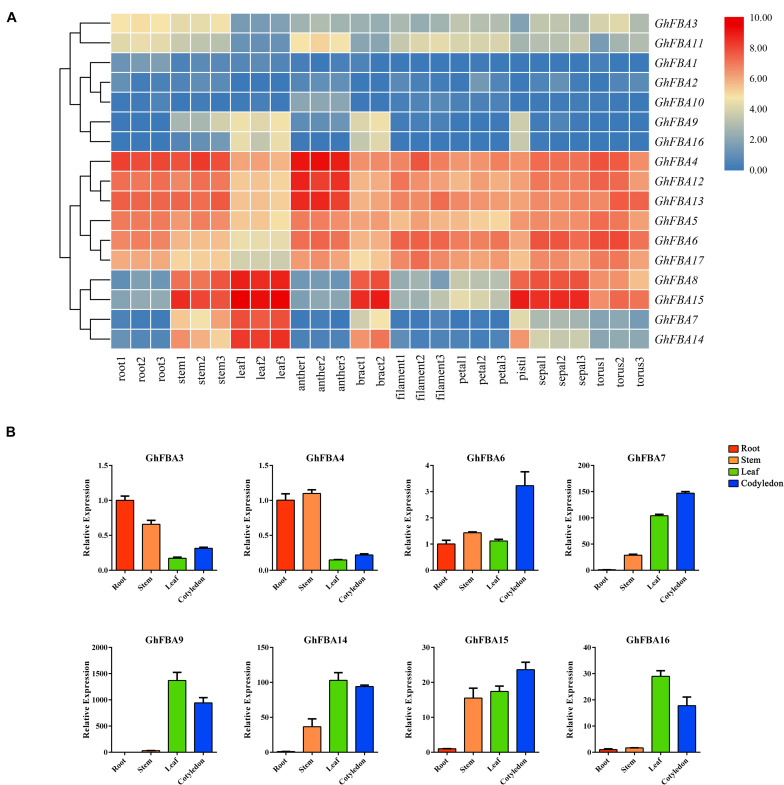
Expression profiles of the *GhFBA* genes in various tissues. **(A)** Hierachical clustering of expression profiles of *GhFBA* genes in various tissues. The TPM values were perform treatment of log_2_(TPM + 1), then the results were used to visualize the heatmap. **(B)** Expression analysis of eight representative *GhFBA* genes in different tissues by qRT-PCR, and *GhHIS* gene was used as a reference gene, vertical bars indicate standard deviation.

To have a further understanding of the functional roles of *GhFBA* members, the expression profiles of *GhFBA* genes in different tissues were analyzed (Figure 6A and Supplementary Table 6). And qRT-PCR experiments were carried out to validate the accuracy and authenticity of the transcriptome data on four representative samples of eight *GhFBA* genes (Figure 6B and Supplementary Table 9). As results, eight genes were commonly (TPM > 1 in all samples) detected in all tissues and four were highly (TPM > 30) expressed. Additionally, some *GhFBA* genes showed very low expression levels in all tissues, such as *GhFBA1*, *GhFBA2* and *GhFBA10*. Besides, some genes displayed significant tissue-specific expression patterns, such as *GhFBA7* and *GhFBA14* were highly expressed in stem, leaf and bract, but *GhFBA8* and *GhFBA15* were highly expressed in stem, leaf, bract, pistil, sepal and torus. In addition, two members of class-II, *GhFBA3* and *GhFBA11*, exhibited low but constitutive expression patterns across the detected tissues.

### Expression Patterns of *GhFBA* Members Under Abiotic Stress Treatments

**FIGURE 7:**
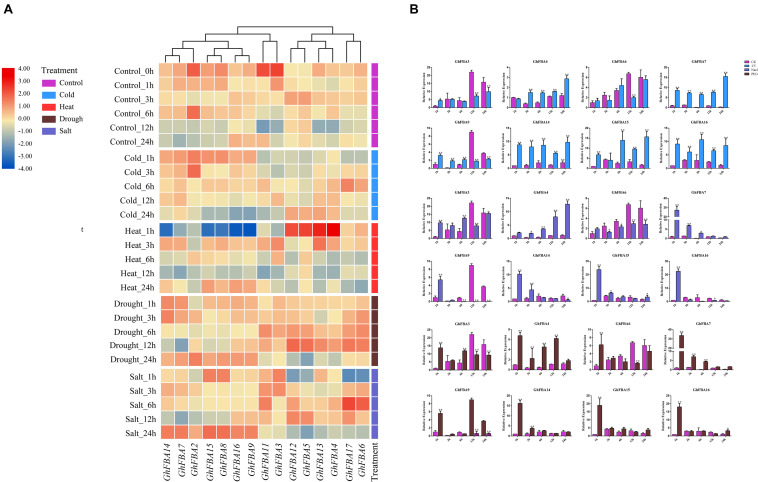
Expression profiles of *GhFBA* genes under different stresses. **(A)** The log_2_(TPM + 1) values of TPM were used to create the heatmap, and z-score method was used to normalize the results by line. The transcript abundances were represented by color scales ranging from blue (low) to red (high). **(B)** Verification of the transcriptome data by qRT-PCR, *GhHIS* gene was used as reference gene, and asterisks indicate the significant differences between treatment groups and control groups (*P < 0.05, **P < 0.01, Student’s *t*-test).

To further analyze the physiological and biochemical functions of *GhFBA* genes under different environment conditions, the expression patterns of *GhFBA* members in response to drought, cold, salt and heat stresses were further investigated according to the transcriptome date (Figure 7A). And the reliability of the transcriptome date was further validate by qRT-PCR based on three samples and eight *GhFBA* genes (Figure 7B). Overall, most numbers of *GhFBA* family were affected by abiotic stresses (Figure 7 and Supplementary Table 7). When treated with cold stress, the expression levels of *GhFBA7/8/14/15/16* were significantly up-regulated. Under heat stress, the expression of *GhFBA7/8/9/14/15/16* was sharply down-regulated at 1 h, but then they were strongly induced at 3 h. Additionally, the expression of *GhFBA4/5/12/13* was up-regulated at 1 h, and gradually returned to normal levels. When treated with drought stress, the expression levels of *GhFBA7/9/14/15* were up-regulated compared to the control groups. Furthermore, under salt stress, the expression levels *GhFBA7/8/9/14/15/16* were up-regulated after 24 h treatment. The expression profiles of *GhFBA11* and *GhFBA17* were not affected by abiotic stress, indicating that they might be house keeping genes. Taken together, *GhFBA* genes displayed various expression patterns in response to adverse environmental conditions, suggesting that *GhFBA* genes might play an important role in stress resistance.

### Expression Analysis of *GhFBA* Genes Under Different Phytohormonal Treatment

**FIGURE 8:**
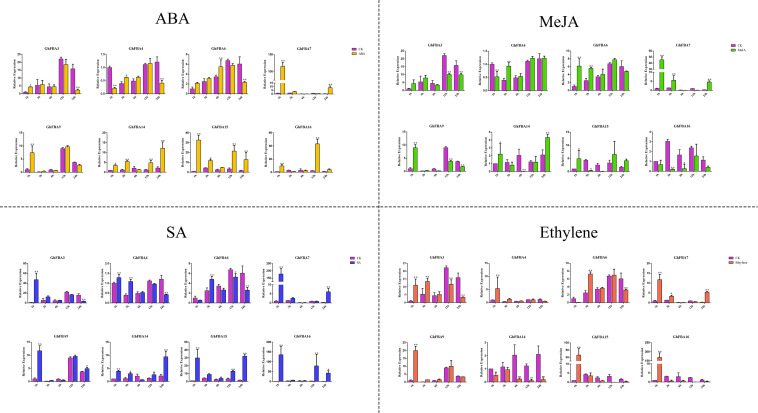
Expression patterns of 8 selected *GhFBA* genes in response to phytohormone treatments. qRT-PCR was performed to analyze the relative expression levels, and *GhHIS* was used as a reference gene. Asterisks indicate the significant differences between treatment groups and control groups (*P < 0.05, **P < 0.01, Student’s *t*-test).

In order to have a further understanding of the regulatory mechanisms of *GhFBA* expression under different phytohormone treatments, 8 *GhFBA* members from different subclasses were selected for qRT-PCR analysis. Overall, phytohormone treatments had a significant effect on the expression levels of the detected genes, but the regulation mechanisms were quite different (Figure 8 and Supplementary Table 8). For instance, *GhFBA3* was significantly induced by SA and ethylene. *GhFBA6* was response to MeJA, SA and ethylene. *GhFBA4/14* showed opposite expression patterns when treated with different hormones, the expression was induced by SA and ethylene but repressed by ABA and MeJA. While the expression of *GhFBA14* was induced by ABA, MeJA and SA, but repressed by ethylene. Moreover, the transient increase of the expression of *GhFBA7/9/15/16* after the phytohormone treatment suggested that these *GhFBA* genes might function as signal molecules in the regulatory pathway of cotton growth and stress tolerance.

### Co-regulatory Networks of *GhFBA* Genes

**FIGURE 9:**
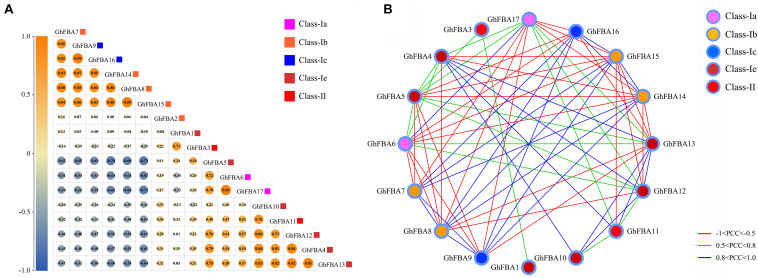
Pairwise correlation and co-regulatory networks of *GhFBA* genes. **(A)** Correlation analysis of *GhFBA* genes. The correlations were based on the PCC values and represented by the size and color of the circles. The squares with different colors were used to represent different subclasses. **(B)** Co-regulatory networks of *GhFBA* gene pairs with | PCC| > 0.5 and *p*-value <0.05. The edge lines with different colors represent the correlation levels of *GhFBA* gene pairs and the nodes with different colors indicate the information of different subclasses.

Based on the public RNA-seq datasets of *G. hirsutum*, the Pearson correlation coefficients (PCCs) were calculated and the co-regulatory networks were constructed. As shown on Figure 9A, the genes within the same subclass usually represent positive correlations, for example, within class-Ia, *GhFBA7*, *GhFBA 8*, *GhFBA14* and *GhFBA15* showed positive correlations with each other. Likewise, two members of class-Ic, *GhGBA9* and *GhFBA16* also exhibited positive correlations. Moreover, positive correlations were observed between the members from different subclasses, such as the members of class-Ia, *GhFBA6/17*, showed positive correlations with *GhFBA4/5/12/13* that belong class-Ie. However, *GhFBA6/17* showed negative correlations with *GhFBA7/8/14/15* and *GhFBA9/16*. Furthermore, all significant PCCs (*p* < 0.05 and | PCCs| > 0.5) of *GhFBAs* were extracted and used to construct the co-regulatory networks. Overall, the co-regulatory networks were constituted with 16 nodes and 70 edges, and only *GhFBA2* showed no correlation with other members. As shown in Figure 9B, 30 *GhFBA* gene pairs showed negative correlations (PCCs < −0.5 and *p*-value < 0.05), 40 gene pairs showed positive correlations and 24 pairs showed strong positive correlations (0.5 < PCCs < 1 and *p*-value < 0.05).

## Discussion

As the initial stage of photosynthetic carbon fixation, Calvin-Benson cycle plays an indispensable role during plant growth and maturation. In recent years, more and more researches suggested that the rubisco was not the only factor that controls photosynthesis rate (Fukayama et al., 2012; Wostrikoff et al., 2012; Feiz et al., 2014; Bracher et al., 2017; Salesse-Smith et al., 2018). *FBA*, as the first bifurcation point of Calvin-Benson cycle, could be a potential target of genetic engineering to increase the photosynthetic carbon CO_2_ fixation rate.

Cotton is not only the main source of renewable textlie fiber, but also an important material for cottonseed oil production. Therefore, as a key enzyme in both photosynthesis and glycolysis (Haritatos et al., 2000; Sasaki and Nagano, 2004). *FBA* genes are of great significance to cotton production. In current study, we totally identified 17 *FBA* genes *G. hirsutum*. The size of *FBA* family in *G. hirsutum* was larger than *A. thaliana* (8) (Lu, 2011), rice (7) and tomato (8) (Cai et al., 2016), but smaller than wheat (21) (Lv et al., 2017) and *B. napus* (22) (Zhao et al., 2019), this result indicated that the number of genes within a *FBA* family might be associated with the genome size. Based on the function domains, *GhFBAs* could be classified into two groups and the members in both groups have the ability to catalyze the hydrolysis of fructose 1,6-bisphosphate (Capodagli et al., 2014). But there was no similarities between them in gene sequence, protein structure or catalytic mechanism, so they are always considered to have developed from different origins (Lebherz and Rutter, 1969; von der Osten et al., 1989; Schnarrenberger et al., 1990; Zgiby et al., 2000). Moreover, we analyzed the *FBA* characteristics in *G. raimondii* and *G. arboreum* (Supplementary Table 1, 9) FBA genes in each species were identified. In theory, the genes of tetraploid *G. hirsutum* should have one-to-one correspondence with the diploid parents, but only 17 *GhFBAs* were found in *G. hirsutum*. A proper explanation of the phenomenon was that after the speciation of *G. hirsutum*, some genes were lost because of the evolutionary divergence between different subgenomes.

The phylogenetic and homologous analysis could provide the information about evolution relationships. Based on the phylogenetic tree, 17 *GhFBAs* members were divided into two classes, and classs-I could be further classified into five subclasses (Figure 2). The members within the same subclass usually shared high similarity in sequences, gene structure and motif composition (Figure 3). Additionally, allopolyploid cotton species, including *G. hirsutum*, appears to have emerged in the last 1-2 million years, so the evolution relationship among these three species are very close. In this study, we totally identified 32 orthologous gene pairs of *FBAs* among the three species (Figure 5). Moreover, gene duplication events played an essential role in forcing the evolution process of genomes and genetic systems (Moore and Purugganan, 2003; Zhu et al., 2014). Segmental, tandem, and transposition events were thought to be the three major patterns (Kong et al., 2007). While in plants, segmental and tandem duplication events were regarded as the primary motivation for the expansion of gene family (Cannon et al., 2004). In this study, 2 segmental duplication evens and one tandem duplication event were identified in *G. hirsutum* (Figure 5), suggesting segmental and tandem duplication events might play important roles in the gene family expansion of *GhFBAs.*

According to the transcriptome date, some valuable information about the potential functions of *GhFBAs* were obtained. *GhFBA4*, *GhFBA5*, *GhFBA6*, *GhFBA12* and *GhFBA13* exhibited constitutive expression patterns in all tissues, which indicated their key roles in the development of *G. hirsutum.* Besides, it’s worth noting that some *GhFBA* members displayed significant tissues-specific expression patterns. For example, the expression of *GhFBA7* and *GhFBA14* were relatively higher in stem and leaf. And *GhFBA8* was specificly expressed in leaf and sepal. High expression levels in green organs suggested that *GhFBA7/14* and *GhFBA8/15* might play important roles in photosynthetic carbon fixation, so they are of great potentials in genetic engineering for improving the photosynthetic efficiency in the future. Furthermore, the diverse expression patterns in different tissues among the members of *GhFBA* family indicated a clear work division of *GhFBA* genes in the growth and development of *G. hirsutum*.

There are considerable evidences that *FBA* genes play important roles in conferring tolerance including cold stress, heat stress, drought stress and high light acclimation (Fan et al., 2009; Lu et al., 2012; Khanna et al., 2014; Oelze et al., 2014; Mu et al., 2021). As the most widespread abiotic stress in field, drought have seriously negative effect on plant growth and development, it usually cause drastically decreases in the yield and quality of crops. Based on the drought transcriptome data, the expression of *GhFBA7/9/14/16* were significantly induced (Figure 7). Further analysis showed that *GhFBA14* have a close phylogenetic relationship with *AtFBA1*, a typical drought-related *FBA* gene in Arabidopsis (Lu, 2011; Lu et al., 2012), suggesting *GhFBA4* possibly plays a critical role in cotton drought stress regulation. Besides drought, temperature is another key factor that affects cotton yield and fiber quality. And it’s noteworthy that the members from class I-e, *GhFBA4/5/12/13*, were all up-regulated when treated with heat stress. Moreover, the complete opposite expression patterns of *GhFBA7/8/9/14/15/16* under heat and cold stresses suggested the their importance in the temperature adaptation. In Arabidopsis, almost all *AtFBAs* were up-regulated under salt stress after 6 h, but only six *GhFBAs* were significantly up-regulated after 24 h. The insensitivity to salt stress indicated a functional difference between *AtFBAs* and *GhFBAs*.

As important signal molecules, phytohormones play crucial roles in regulating the plant growth and stresses resistance. However, only a few literature suggested that the expression of *FBA* genes were regulated by phytohormones (Konishi et al., 2004; Tanaka et al., 2004; Osakabe et al., 2005). According to the qRT-PCR results, all selected genes were significantly affected by phytohormone, but the expression patterns were quite different. And these complex regulation networks indicated that *GhFBA* genes participate in multiple biological process during plant growth through phytohormones pathways in cotton. Furthermore, the expression of *GhFBA7, GhFBA9, GhFBA15* and *GhFBA16* was immediately up-regulated at 1 h after the treatment and then return to the normal levels quickly within 3 h. Based on these dramatic changes of the expression, it could be hypothesized that *GhFBA7/9/14/16* might act as signal molecules in cotton phytohormone signal transduction, and further experiments were needed to verify this theory.

According to the expression analysis, most *GhFBA* members have one or more partners with similar expression patterns. Together with the result that *AtFBA* single mutant always showed no phenotypic variation (Lu, 2011), we conjectured that cotton *FBA* genes are functionally redundant. As an indispensable part of glycolysis and Calvin-Benson cycle, the existence of multiple members with similar functions ensures the normal metabolism of cell and eventually lead to the redundancy of *FBA* members. Furthermore, most *GhFBAs* were response to multiple phytohormone and stress treatments, suggesting there is a remarkable function crosstalk and work division among the members. The redundancy of *GhFBA* genes ensure the normal growth when some members lose their function, and the work division allow cotton to quickly respond and adapt to new environment when living conditions change.

In *Brassica napus* and *Triticum aestivum* (Lv et al., 2017; Zhao et al., 2019), the *FBA* genes showed close correlations according to the expression patterns and could be classified into different clusters. While in *G. hirsutum*, the co-regulatory networks were more complex and hardly to be classified (Figure 9B). The origin of *G. hirsutum* might be a key factor for this results. In the diploid parents of *G. hirsutum*, two complex regulatory networks of *FBA* genes have already existed, after the interspecific hybridization, two networks interacted with each other and eventually formed a more complicated co-regulatory network of *GhFBA* genes. Additionally, the complexity of co-regulatory networks further indicated the coexistence of work division and functional redundancy between *GhFBA* genes.

Overall, current study performed a genome-wide analysis of *FBA* gene family in *G.hirsutum* for the first time. And the results will shed light on the functional analysis of *GhFBA* genes and provide valuable resources to deeply explore their biological roles in improving cotton yield and quality.

## Data Availability Statement

The datasets presented in this study can be found in online repositories. The names of the repository/repositories and accession number(s) can be found in the article/Supplementary Material.

## Author Contributions

Z-QL and YZ: bioinformation analysis and data processing. HL, T-TS, C-GL, and Z-CH: cotton plants cultivating, material treatment and sample collection. Z-QL, HL, and T-TS: RNA extraction and qRT-PCR experiment performing. Z-QL: manuscript writing. YZ: discussion writing and manuscript review. All authors contributed to the article and approved the submitted version.

## Conflict of Interest

The authors declare that the research was conducted in the absence of any commercial or financial relationships that could be construed as a potential conflict of interest.

## Publisher’s Note

All claims expressed in this article are solely those of the authors and do not necessarily represent those of their affiliated organizations, or those of the publisher, the editors and the reviewers. Any product that may be evaluated in this article, or claim that may be made by its manufacturer, is not guaranteed or endorsed by the publisher.

## Footnotes

1
https://cottonfgd.org

2
http://pfam.xfam.org

3
http://smart.embl-heidelberg.de/

4
https://web.expasy.org/protparam/

5
http://gsds.cbi.pku.edu.cn

6
http://www.genscript.com/wolf-psort.html

7
http://meme.nbcr.net/meme/intro.html

8
https://github.com/CJ-Chen/TBtools

## Supplementary Material

Supplementary Table 1 Primers used in qRT-PCR analysis.

Supplementary Table 2 Characteristics information of *FBA* family genes in *G. raimondii* and *G. arboreum*.

Supplementary Table 3 Annotation of cis-acting regulatory elements in the promoters of *GhFBA* genes.

Supplementary Table 4 Gene duplication of *FBA* gene in Gossypium hirsutum.

Supplementary Table 5 One-to-one orthologous relationships between *G. hirsutum* and other two cotton species.

Supplementary Table 6 The public available RNA-seq date of *GhFBA* genes in different tissues.

Supplementary Table 7 The public available RNA-seq date of *GhFBA* genes under different stress treatments.

Supplementary Table 8 Expression profiles of 8 *GhFBA* genes in response to different stress and phytohormone treatments.

Supplementary Table 9 Expression patterns of 8 *GhFBA* genes in four different tissues.

## Abbreviations

Rubisco: Ribulose-1,5-bisphosphate carboxylase/oxygenase
RuBP: ribulose-1,5-bisphosphate
FBP: fructose-1,6-diphosphate
G3P: glyceraldehyde-3-phosphate
DHAP: dihydroxyacetone phosphate
SBP: sedoheptulose 1,7-bisphosphate
E4P: erythrose 4-phosphate
cFBA: cytosolic located FBA
cpFBA: chloroplast/plastid located FBA
TPM: transcripts per million
qRT-PCR: quantitative real-time PCR
ABA: abscisic acid
SA: salicylic acid
MeJA: Methyl Jasmonate.
